# Human Disc Degeneration Is Accompanied by a Loss of Anterior Annulus Fibrosus Interlamellar Matrix Integrity as Assessed by Peel Tests

**DOI:** 10.1002/jsp2.70067

**Published:** 2025-05-14

**Authors:** Manmeet S. Dhiman, Mohammed A. Salaam, Taylor J. Bader, Fred Nicholls, W. Bradley Jacobs, Kenneth C. Thomas, Jacques Bouchard, Paul T. Salo, David A. Hart, Ganesh Swamy, Neil A. Duncan

**Affiliations:** ^1^ McCaig Institute for Bone and Joint Health University of Calgary Calgary Alberta Canada; ^2^ Department of Biomedical Engineering University of Calgary Calgary Alberta Canada; ^3^ Department of Medical Sciences University of Calgary Calgary Alberta Canada; ^4^ Department of Surgery, Cumming School of Medicine University of Calgary Calgary Alberta Canada; ^5^ Faculty of Kinesiology University of Calgary Calgary Alberta Canada; ^6^ Department of Civil Engineering University of Calgary Calgary Alberta Canada

**Keywords:** annulus fibrosus, degenerative disc disease, interlamellar cross‐bridges, interlamellar matrix, interlamellar peel mechanics, intervertebral disc, low Back pain

## Abstract

**Introduction:**

Disc degeneration (DD) is accompanied by biomechanical changes in the intervertebral discs. The lamellae of the annulus fibrosus (AF) are interconnected through the interlamellar matrix (ILM). The ILM contains interlamellar cross‐bridges, connecting the lamellae radially in three dimensions. Weakening of the ILM and the cross‐bridges could contribute to delamination between the lamellae, reducing their ability to resist loads and thus contributing to loss of AF integrity associated with the development and progression of degeneration. The objective of the present study was to quantify the differences in interlamellar mechanical properties of fresh AF samples from surgical DD individuals compared to AF samples from non‐DD donors.

**Methods:**

An interlamellar peel test was performed on fresh AF tissue collected from DD surgeries (*n* = 36) and non‐DD organ donors (*n* = 13). The tissue was peeled at 0.5 mm/s until complete separation. Interlamellar mechanical properties were calculated from the force‐displacement curve.

**Results:**

Samples from DD individuals had lower Peel Stiffness (*p* = 0.001), Peel Strength (*p* = 0.001), Peel Toughness (*p* = 0.0009), and Standard Deviation of the Peel Stress (*p* = 0.02) compared to the tissue from non‐DD organ donors. Age had moderate negative correlations with Peel Stiffness (*R* = −0.59), Peel Strength (*R* = −0.66), and Peel Toughness (*R* = −0.69) for non‐DD samples only.

**Discussion:**

The mechanical integrity of the ILM was determined to be lower in surgical DD individuals compared to non‐DD donors. Aging alone may not have affected the results, and rather, loss of the integrity of ILM during disease progression appeared to have significantly contributed to the differences observed. This study provides new mechanical insights into the delamination often observed in the AF of surgical DD individuals. Future biochemical and immunolocalization studies, integrated with mechanical data, will aim to understand the role of collagen and elastin structure and composition in the decreased mechanical integrity of affected tissues.

## Graphical abstract

Disc degeneration (DD) is associated with reduced mechanical integrity of the interlamellar matrix (ILM) in the annulus fibrosus (AF), as demonstrated by significantly lower Peel Stiffness, Peel Strength, Peel Toughness, and Standard Deviation of the Peel Stress in the Peel Region in DD samples compared to non‐DD samples. Aging alone may not explain these differences, suggesting that disease progression is critical in ILM weakening and delamination. This study provides new insights into AF degeneration mechanics and highlights the need for future biochemical research to explore the contributions of collagen and elastin to ILM integrity.

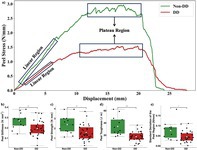

## 1 Introduction

Low back pain (LBP) is reported to affect greater than 40% of adults in the United States and the Western world at some point in their lives and is a leading cause of disability with major socio‐economic impact, with up to 33% of the patients being unable to work for about 100 days per year [1, 2]. LBP is associated with disc degeneration (DD) [3, 4, 5, 6]. However, a clear mechanism and the degree of involvement of various structures of the intervertebral disc (IVD) in the development of DD remain elusive.

The IVD consists of the gelatinous nucleus pulposus (NP), the surrounding lamellar structure of the annulus fibrosus (AF), and the cartilaginous end plates [1, 7, 8]. The lamellae of the AF are interconnected through connective tissue called the interlamellar matrix (ILM) [9]. The ILM is composed of macromolecules such as collagen type VI [10, 11], proteoglycans [12], and elastin [13, 14], and contains interlamellar cross‐bridges that primarily interconnect the annular lamellae in three dimensions [15]. The cross‐bridges also contain anionic proteoglycans, which may help prevent damage to the ILM by allowing more sliding movement of the collagen bundles relative to each other during loading [16, 17, 18]. A dense network of elastin fibers surrounds the collagen bundles along the lamellae and across the ILM [13, 19], which may provide a restoring force following mechanical deformation due to its proximity to the collagen bundles. Some collagen Type I can also be detected in the ILM, but there is no detectable collagen type II, IX, or X, unlike other structures in the IVD (e.g., inner AF and NP) [10]. The location and the components of the ILM suggest a vital role in preventing delamination, and further investigation into its mechanical role is required.

As NP volume decreases, vertical compressive stresses are redistributed to the AF, resulting in the inner AF bulging radially with reduced intradiscal pressure [20]. The AF becomes more disorganized, with irregular elastic fibers and randomly oriented collagen bundles [21], making the AF more susceptible to structural failure. Annular tears are categorized as circumferential tears (delamination), peripheral rim tears, and radial fissures [22, 23], with circumferential tears potentially resulting from abnormal interlamellar shear stresses [24]. Although numerous studies have explored the tensile, shear, and compressive properties of the AF in multiple directions and planes [25, 26, 27, 28, 29, 30], the contributions of the ILM and cross‐bridges to the gross mechanics of the AF and their relationship to the development and progression of DD have been less extensively reported in the literature. The ILM has been shown to be the weaker structure in disc herniations compared to the lamellae [31]. Computational and experimental models have shown that the ILM plays an important role in circumferential, radial, and axial directions during uniaxial and biaxial loading for viscoelastic and elastic properties [32, 33]. Furthermore, the isolated elastic fiber network of the ILM exhibits non‐linear elastic behavior and plays a vital role in the tensile and shear behavior of the ILM [34].

To quantify the characteristics of the ILM and its cross‐bridges to AF mechanics, Gregory et al. described an interlamellar peel test [35, 36, 37, 38, 39, 40] to directly measure adhesive strength between lamellae. They found a 33% increase in Peel Strength for the outer AF samples compared to the inner AF samples harvested from frozen human cadaveric tissues (Pfirrmann grade 2 or 3 on the 1–5 scale) [37]. These authors subsequently used an annular puncture model to induce degeneration in a New Zealand white rabbit model [38] and reported that the puncture group had a 30% decrease in Peel Strength compared to the control group. To date, the peel test has not been used in the exploration of human pathological conditions and comparisons of samples with different degeneration grades.

For the present study, it was hypothesized that the interlamellar properties would be reduced in outer AF samples obtained at surgery for DD compared to non‐DD samples. The purpose of the study was to quantify the differences in interlamellar mechanical properties of fresh AF samples from surgical individuals with DD compared to non‐DD AF samples from organ donors.

## 2 Methods

### 2.1 Inclusion/Exclusion Criteria and Ethics

**FIGURE 1:**
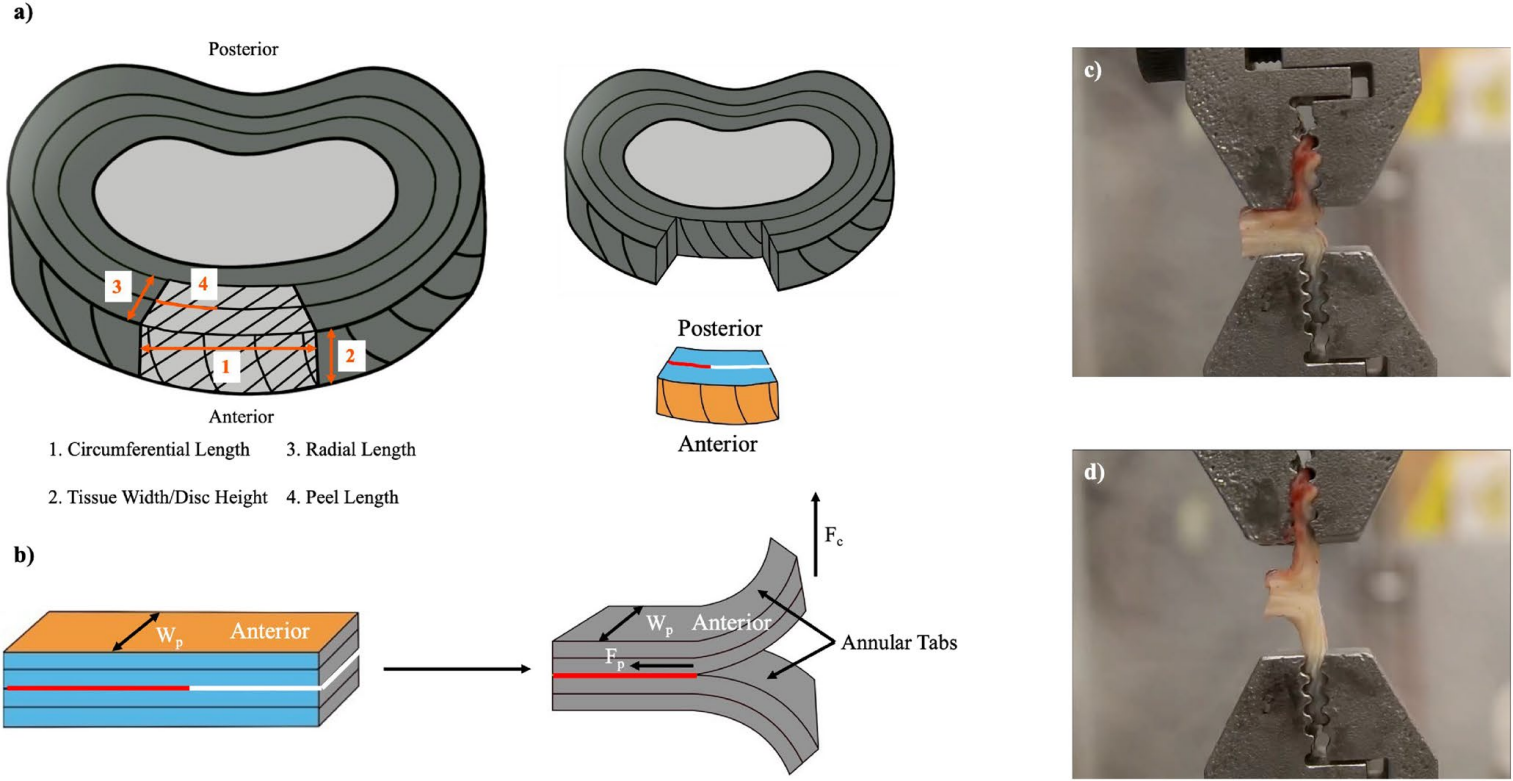
(a) Schematic of the AF sample harvested (light shaded portion) from the individuals with degenerative disc disease and non‐DD individuals. Sample dimension parameters labeled as: (1) Circumferential Length, (2) Disc Height/Tissue Width, (3) Radial Length, and (4) Peel Length. The blue and orange shaded faces of the sample showcase the change in orientation of the sample from (a) to (b). (b) Schematic showing the preparation of the peel test sample. The tissue strip on the left shows the Peel Length in red and the projected incision in white. The tissue is cut along the white line using a number 11 scalpel blade. The tissue strip on the right with the incision shows the annular tabs that are used to clamp the tissue for the peel test. *W*_p_ is the tissue width (disc height), *F*_p_ is the force experienced by the tissue for delamination, and *F*_c_ is the force exerted by the clamp to stretch and delaminate the tissue. Clamp force, *F*_c_, is assumed to be equal to the peel force, *F*_p_. (c) The AF sample is mounted onto the clamps before starting the peel test. (d) The AF sample is peeling along the incision made during sample preparation until complete separation.

Fresh human AF samples were tested within 60 min after removal from individuals undergoing anterior lumbar surgery for DD at L4‐L5 and L5‐S1 levels. Non‐DD fresh samples were obtained from organ donors through the Southern Alberta Tissue Donation program and tested immediately after organ retrieval within 1–2 h of death. All samples (both non‐DD and DD) were obtained with local ethics approval (University of Calgary Ethics ID: REB18‐1308) from individuals with no overt spine deformities or any other known connective tissue disorders. Samples with minimum dimensions of 15 mm × 3 mm × 2 mm (circumferential length × tissue width (disc height) × radial length) were included (Figure 1a).

### 2.2 Demographics

In Table 1, BMI and modified Pfirrmann Grade [41] were not available for the non‐DD donors, although the discs sampled appeared grossly healthy and none of the donors were morbidly obese. The radiographic grading system by Wilke et al. [42] was used to grade samples from 6 of 7 non‐DD donors on lateral computed tomography (CT) scans (images available in Dhiman et al. Supporting Information Section S1 [43]), as radiographs and magnetic resonance imaging (M.R.I.) were not available. Macro visual inspection also did not reveal any significant degenerative changes in non‐DD donors. Only M.R.I. was available for pre‐operative imaging of DD individuals; thus, the modified Pfirrmann Grade was recorded only for the DD individuals. For the non‐DD group, discs from both L4‐L5 and L5‐S1 levels were obtained, except for one individual where only the L4‐L5 disc was tested. For the DD group, both L4‐L5 and L5‐S1 discs were obtained from 8 individuals, only L4‐L5 discs from 6 individuals, and only L5‐S1 discs from 14 individuals.

**TABLE 1:** Basic demographic information of the Non‐DD and DD individuals included in the study.

	Non‐DD	DD
Number of individuals	7	28
Number of samples	13	36
Age (years)*	35 ± 9	44 ± 10
BMI	N/A	26.7 ± 3.3
Modified Pfirrmann grade	N/A	6.5 ± 1.3
Radiographic degeneration grade	0.25 ± 0.45	N/A
Sex (M/F)	(5/2)	(18/10)
Disc level (L4‐L5/L5‐S1)	(7/6)	(14/22)

*Note:* *p ≤ 0.05 for the comparison of age between non‐DD and DD individuals. Age, body mass index (BMI), and sex distribution are reported by the number of individuals whereas the modified Pfirrmann Grade [41], radiographic degeneration grade [42], and disc level distribution are reported by the number of samples.

### 2.3 Tissue Preparation and Mechanical Testing

An incision (length of the circumferential length minus the peel length) was made at one end of each AF sample in the circumferential direction to create annular tabs and a T‐shaped sample (Figure 1b). The four tissue dimensions (Figure 1a) were measured using a digital caliper. Due to the heterogeneity in the size of the tissue samples obtained, standardizing all tissue dimensions was not feasible except for peel length. The peel length was standardized to 8–9 mm. Fresh samples were mounted on a 2.2 KIP Capacity MTS Model 242.02 Universal test frame (MTS Systems Corporation, Minnesota) with a hydraulic actuator and a 100 N (± 0.5 N) MTS load cell. Custom‐built clamps were utilized to mount the tissue for peel testing (Figure 1c). After mounting, the tissue was exposed to 100% water vapor relative humidity at room temperature and allowed to reach equilibrium in the clamps for 10 min before starting the test [25]. The tissue was exposed to a humidifier instead of immersing it in PBS due to concerns about the impact of osmolarity on mechanical properties [44]. The tissue underwent 20 cycles of preconditioning (to verify force stabilization, ensuring that viscoelastic effects did not influence our measurements) at 0.5 Hz with 0.25 mm positive cyclic displacement to ensure adequate sample placement in the grooves of the clamps, followed by 5 min of stress‐relaxation. The tissue was then peeled at 0.5 mm/s (chosen as the medium delamination rate to minimize time‐dependent effects that may have occurred at 0.05 mm/s (slow rate) or 5 mm/s (fast rate)) until complete separation (approximately 1–2 min duration) was achieved [37, 39] (Figure 1d). A force‐displacement‐time curve was generated by the MTS Universal Testing machine, and the data were sampled at 100 Hz. The force‐displacement curve was normalized to tissue dimensions by dividing the peel force by the tissue width (Equation 1).

(1) $$\sigma_{p}\;\left(\text{Peel Stress}\right)=\frac{F_{c}\;\left(\text{Peel Force}\right)}{W_{p}\;\left(\text{Tissue Width}\right)}\;\left(\frac{N}{\mathrm{mm}}\right)$$

**FIGURE 2:**
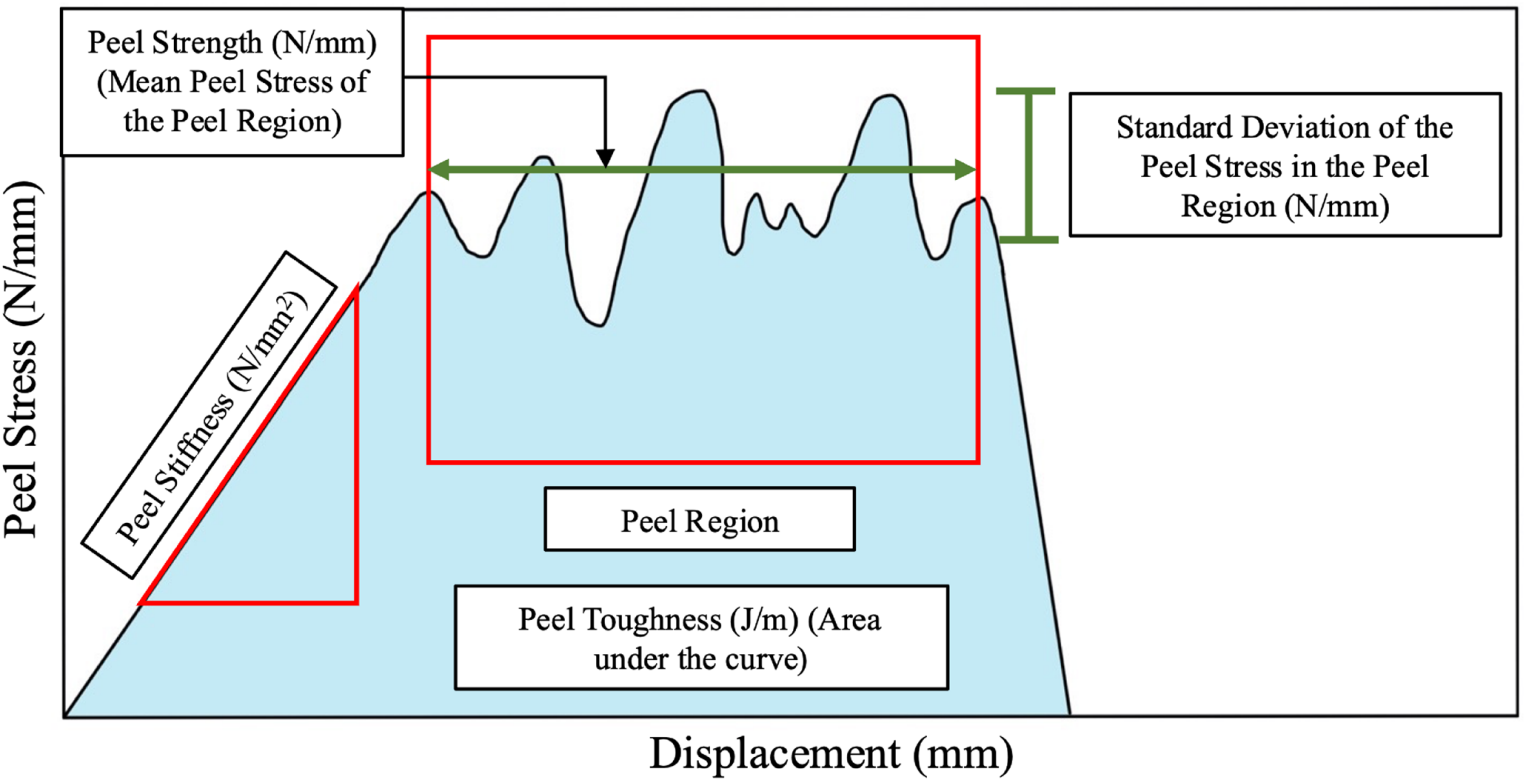
A schematic curve from a peel test of the AF. Peel Stiffness is the slope of the initial linear region. Peel Strength is the mean Peel Stress of the flatter Peel Region. Peel Toughness is the total area under the curve. Standard Deviation of the Peel Stress in the Peel Region is the standard deviation of the Peel Stress isolated using the user‐defined Peel Region.

Peel Stress is a result of one‐dimensional normalization (tissue width only) whereas force is usually normalized two‐dimensionally (cross‐sectional area) to obtain the stress in tensile, shear, compressive, and torsional stress calculations. Thus, the units of stress are different (N/mm versus N/mm^2^ or MPa) in this case. Since strain is not meaningful to be defined, clamp displacement is on the *x*‐axis, which means peel stiffness and strength defined below also have non‐standard units. A custom Python script was developed to analyze the peel stress‐displacement curve (Figure 2) and calculate the following parameters: Peel Stiffness (N/mm^2^): resistance to initiate delamination, Peel Strength (N/mm): maximum strength of the cross‐bridges and the matrix to prevent delamination, Peel Toughness (J/m): energy required for complete delamination, and Standard Deviation of the Peel Stress in the Peel Region (N/mm): an indirect measure for strength of individual cross‐bridges. As per the criteria set by Briar et al. [39], the start and end of the Peel Region is defined such that the mean Peel Stress value remains relatively stable (a plateau region) after the curve first becomes nonlinear following the initial linear region. Similarly, Peel Stiffness was also calculated by identifying the linear region based on the criteria set by Briar et al. [39]. A rolling linear line of best fit was calculated for multiple bounds, and the slope from the line with the highest *R* ^2^ was selected (minimum *R* ^2^ = 0.95).

### 2.4 Statistics

Shapiro–Wilk normality tests and *F*‐tests were performed to check for normality and differences between variances of the non‐DD and DD groups before conducting statistical tests. One‐tailed Student's *t*‐tests and Welch's *t*‐tests were performed to test for significance given the directionality of the hypothesis based on previous animal data [38]. The Type I error was set to *α* = 0.05. Correlation coefficients were calculated for the four interlamellar parameters with respect to age for both non‐DD and DD samples using the Generalized Estimating Equations (GEE) approach. Correlation coefficients using the same approach were also calculated between the four interlamellar properties for both non‐DD and DD samples.

## 3 Results

**FIGURE 3:**
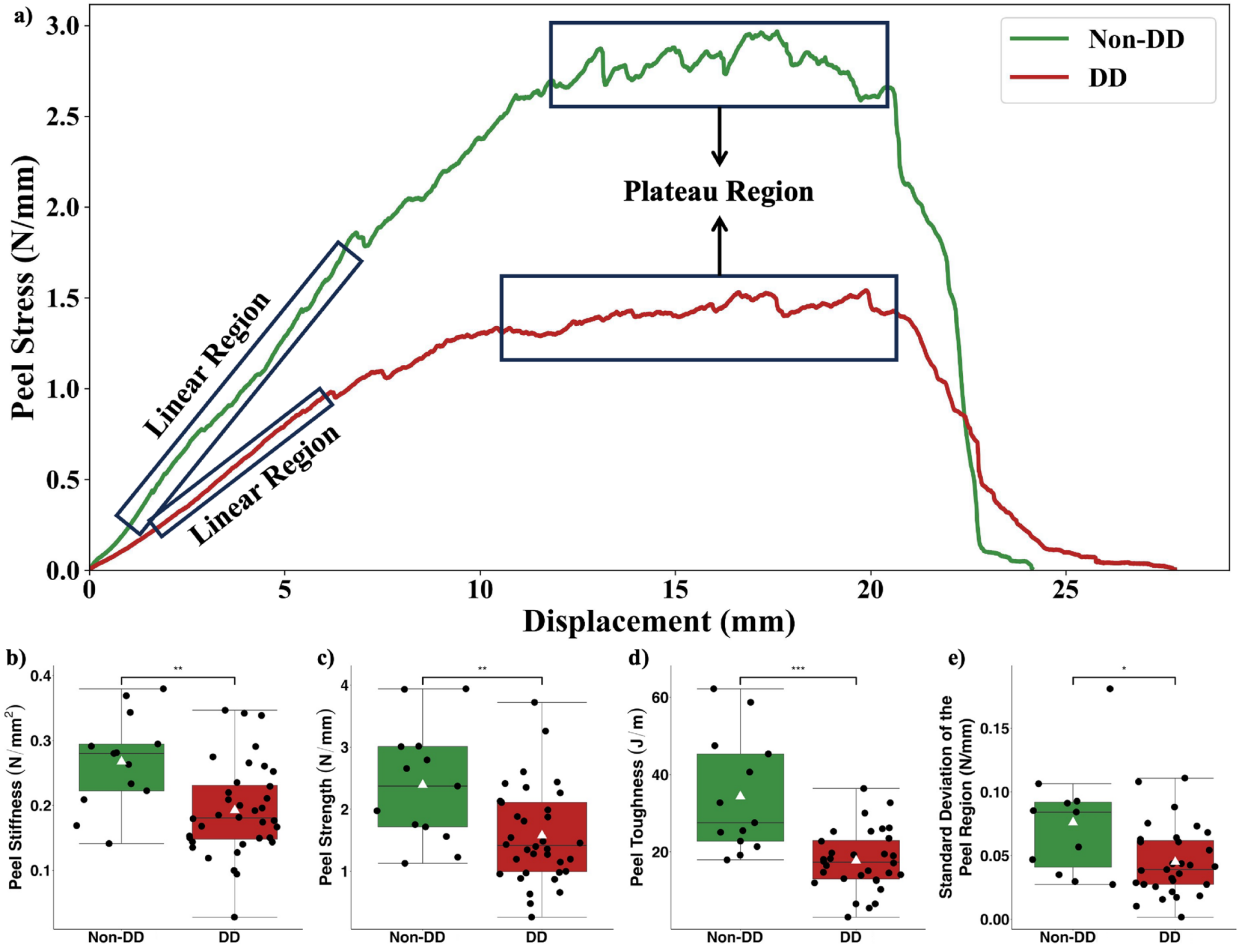
(a) Representative mean peel curves for the non‐DD (green) and DD (red) samples. The linear region used to calculate the Peel Stiffness, and the plateau region (Peel Region) used to calculate the Peel Strength and Standard Deviation of the Peel Stress are also defined for the non‐DD and DD samples. Comparison of the (b) Peel Stiffness, (c) Peel Strength, (d) Peel Toughness, and (e) Standard Deviation of the Peel Stress in the Peel Region between non‐DD and DD samples. The horizontal line in the middle of the boxplot represents the median and the white triangle represents the mean of the group. ***p ≤ 0.001, **p ≤ 0.01, and *p ≤ 0.05.

The peel stress‐displacement curve for all samples showed an expected linear increase in peel stress, followed by a plateaued peel region, and then a subsequent decrease (Figure 3a). One non‐DD sample and four DD samples were excluded due to slippage. Three DD samples were excluded due to deviation of the peeling from the incision line during the test. Both slippage and deviation were confirmed by video recordings of the tests.

### 3.1 Assessment of ILM Characteristics: Comparisons of DD Samples With Non‐DD Samples

Tissue obtained from the DD individuals had lower Peel Stiffness (*p* = 0.001), Peel Strength (*p* = 0.001), Peel Toughness (*p* = 0.0009), and Standard Deviation of the Peel Stress in the Peel Region (*p* = 0.02) compared to the samples obtained from non‐DD individuals (Figure 3b–e) (Table 2).

**TABLE 2:** Summary of the comparison of interlamellar peel properties between non‐DD and DD individuals.

	Non‐DD	DD
Peel stiffness (N/mm^2^)	0.27 ± 0.07	0.19 ± 0.07**
Peel strength (N/mm)	2.39 ± 0.93	1.58 ± 0.76**
Peel toughness (J/m)	34.33 ± 15.05	17.74 ± 7.79***
Standard deviation of the peel region (N/mm)	0.076 ± 0.045	0.045 ± 0.027*

*Note:* Values are reported as mean ± standard deviation. ***p ≤ 0.001, **p ≤ 0.01, and *p ≤ 0.05 for statistical differences between non‐DD and DD samples.

### 3.2 Effect of Aging

**FIGURE 4:**
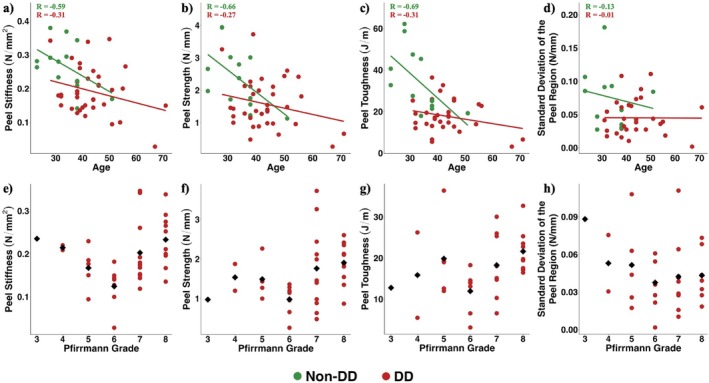
Correlation coefficients were calculated with respect to age and (a) Peel Stiffness, (b) Peel Strength, (c) Peel Toughness, and (d) Standard Deviation of the Peel Stress in the Peel Region for the non‐DD (green) and DD (red) samples. All coefficients are calculated using the GEE approach based on the normality of the data. Means of each Pfirrmann Grade for DD samples from 3 to 8 (black diamonds) were calculated for (e) Peel Stiffness, (f) Peel Strength, (g) Peel Toughness, and (h) Standard Deviation of the Peel Stress in the Peel Region for DD samples only.

Age had moderate negative correlations with Peel Stiffness (*R* = −0.59), Peel Strength (*R* = −0.66), and Peel Toughness (*R* = −0.69) in non‐DD samples (Figure 4a–c). However, age was weakly correlated with the three parameters in DD samples (Peel Stiffness: *R* = −0.31, Peel Strength: *R* = −0.27, and Peel Toughness: *R* = −0.31). Age was not correlated in both non‐DD and DD samples with Standard Deviation in the Peel Region (Non‐DD *R* = −0.13 vs. DD *R* = −0.01) (Figure 4d).

### 3.3 Relationship With Pfirrmann Grade

Peel Stiffness, Peel Strength, and Peel Toughness exhibited a declining trend from Pfirrmann Grade 4–6, after which they showed an upward trend from Grade 6–8 among DD samples (Figure 4e–g). However, no such trend was observed for the Standard Deviation of the Peel Region among DD samples (Figure 4h).

### 3.4 Correlations Among Interlamellar Parameters

**FIGURE 5:**
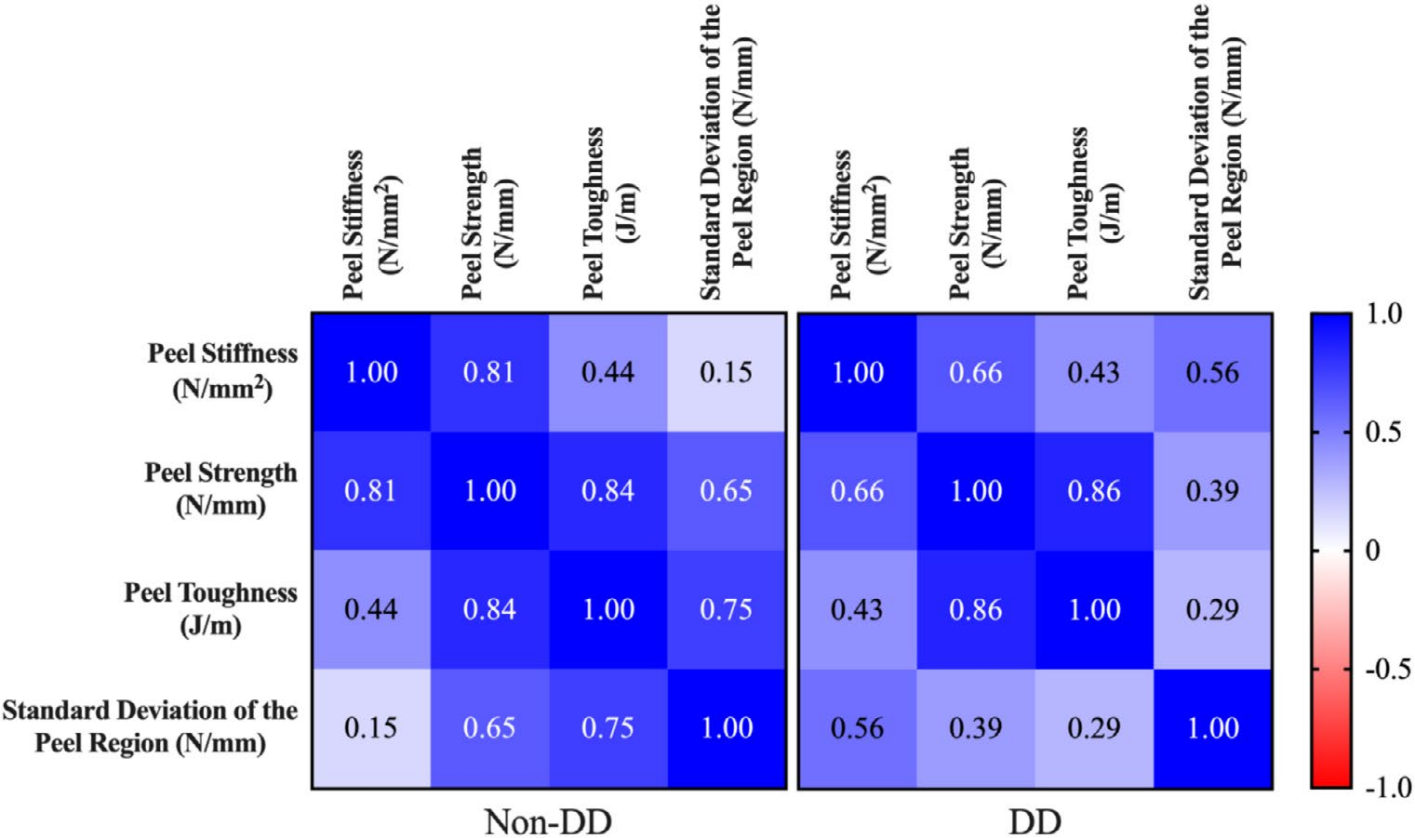
Correlation coefficients for the relationships between the four interlamellar parameters for both non‐DD and DD samples. All correlation coefficients are calculated using the Generalized Estimating Equations (GEE) approach.

Samples from non‐DD donors had strong positive correlations between Peel Stiffness and Peel Strength (*R* = 0.81), Peel Strength and Peel Toughness (*R* = 0.84), and Peel Toughness and Standard Deviation of the Peel Region (*R* = 0.75) (Figure 5). For non‐DD samples, moderate positive correlations were observed between Peel Stiffness and Peel Toughness (*R* = 0.44) and Peel Strength and Standard Deviation of the Peel Region (*R* = 0.65). Among DD samples, a strong correlation was observed between Peel Strength and Peel Toughness (*R* = 0.86) (Figure 5). Moderate positive correlations were observed between Peel Stiffness and Peel Strength (*R* = 0.66), Peel Stiffness and Peel Toughness (*R* = 0.43), and Peel Stiffness and Standard Deviation of the Peel Region (*R* = 0.56) for DD samples.

## 4 Discussion

Several interlamellar mechanical properties of the outer AF samples obtained from DD individuals differed from those obtained from non‐DD donors. The data suggest that the adhesion between the lamellae is decreased in the outer AF of the DD disc samples. A previous study by Gregory et al. also reported decreased Peel Strength in the AF samples from a rabbit annular disc puncture model used to induce degeneration from acute injury [38]. Their study reported direct evidence for the link between DD and Peel Strength, a measure of resistance to delamination. Combined with the present study with the human AF samples, DD is associated with a reduced lamellar adhesion strength, which may be a factor in AF delamination, a sign of degenerative changes in the IVD [22].

The samples from non‐DD individuals exhibit minimal delamination compared to the surgical samples from individuals with DD based on the review of DD by Adams and Roughley [22], and the current dataset is in agreement. Because of degeneration, loss of NP volume leads to a reduction in disc height and increased compressive loads in the AF, which radially deforms the AF outwards and inwards. This results in increased tensile radial strains, which may lead to an increased risk for delamination. Healthy IVDs do not experience a similar loss of pressure in the NP and thus may not experience the tensile radial strains to the same extent as such degenerated IVDs. Such increased strains may be related to the decreased structural strength of the ILM during disease progression in DD [45].

The previous study by Gregory et al. [38] exploring the effect of degeneration on the ILM was conducted in rabbits, and no studies have been reported to explore the analogous degenerative changes in human tissue. Another previous study by Gregory et al. examined the Peel Stiffness and Strength of relatively healthy frozen AF tissue (original Pfirrmann Grade 2 and 3) from human lumbar spines, and they reported the methodological optimization of the peel test [37]. The mean Peel Stiffness from the anterior AF of the non‐DD samples in the current study (0.27 N/mm^2^) was similar to the anterior non‐DD samples in the study by Gregory et al. (~0.30 N/mm^2^). However, Peel Strength in degenerative samples was significantly lower in their study with human samples with Pfirrmann grade of 2–3 on a scale of 1–5. The current study recorded a mean Peel Strength of 2.39 N/mm and 1.58 N/mm for the non‐DD and DD groups, respectively, whereas the study by Gregory et al. [37] reported a mean Peel Strength of ~0.60 N/mm from the outer anterior AF region of frozen human DD samples. The basis for these study differences may be due to the use of fresh and frozen samples and/or the use of external hydration in the current study but remains to be elucidated.

Parameters related to Peel Stiffness and Strength can also provide novel insights into the behavior of the individual cross‐bridges. Standard Deviation of the Peel Stress in the Peel Region was recently explored by Briar et al. in healthy bovine AF samples to examine the differences between peel rates [39]. Although a direct species comparison is not possible, they reported ~0.50 ± 0.19 N/mm with the same displacement rate as the current study (0.5 mm/s), which is 7‐fold higher than the present study for non‐DD human samples (0.076 ± 0.045 N/mm). The difference may be due to the total peel length of the samples (40 mm in their study vs. 8 mm in the present study) as greater sample length increases the probability of breaking more bridges with varying strength, leading to higher Standard Deviation of the Peel Stress. The standard deviation indirectly represents the strength of the cross‐bridges and will vary by bridge strength. A strong bridge will result in a large peak and valley in the peel stress, thus increasing the standard deviation. Weaker bridges will have small deflections while breaking, and thus a smaller standard deviation would be expected. Thus, the lower Standard Deviation of the Peel Stress in the Peel Region (0.046 N/mm) in DD samples compared to the non‐DD samples (0.076 N/mm) is supportive of reduced interlamellar strength in the DD individuals. Peel Toughness was also determined to be lower in the samples from DD individuals compared to those from the non‐DD donors. The results indicate that the DD samples delaminate more readily with lower energy, which further suggests a lower mechanical integrity of the ILM.

The Peel Stiffness, Peel Strength, and Peel Toughness were moderately correlated with age for the non‐DD samples, whereas no such trend was detected for DD samples. It has been challenging to differentiate between changes resulting from physiological aging and those associated with a disease process [46, 47, 48]. The weak correlations between age and interlamellar properties of the DD samples suggest that the differences observed in interlamellar properties between the non‐DD and DD groups are less likely due solely to age; rather, the disease progression is likely a contributing factor adding to the total alterations observed in tissue integrity and matrix turnover [43]. Additionally, a brief age‐matched analysis (Section S1) using the “MatchIt” R package [49] still showed similar differences between the non‐DD and DD samples.

The Peel Stiffness, Peel Strength, and Peel Toughness had increasing trends with the modified Pfirrmann Grade (Grade 6–8) for DD samples. However, the variability observed in the interlamellar properties among the non‐DD samples would not be explained by their consistently low degeneration profile (Table 1). The Pfirrmann Grade represents the degeneration of the IVD, which has been associated with a decrease in radial tensile [26] and circumferential tensile [50] properties. Since the radial tensile test may be associated with the interlamellar properties due to its set‐up, a similar negative correlation was expected using the peel test. The trends observed in the current study with an increase in some mechanical properties at higher Pfirrmann grades may be due to the biological adaptation of the AF in an attempt to preserve its functional mechanical properties.

The different grading schemes for the non‐DD and DD groups also make it challenging to interpret the differences between the two groups. However, the common parameter between the two grading scales is the disc height. Using the radiographic degeneration grading scale for comparing disc height, most DD discs would be scored either a 2 or a 3 (on a scale of 0–3) whereas all non‐DD discs had a score of 0 (no disc height loss). So, DD discs were significantly more degenerated compared to the non‐DD discs.

All four reported parameters in the current study (Peel Stiffness, Peel Strength, Peel Toughness, and Standard Deviation of the Peel Region) are measures of the mechanical integrity of the ILM. The results suggest that the parameters for the mechanical integrity have strong or moderate positive correlations with each other for both non‐DD and DD samples. The moderate to strong correlations between the interlamellar properties add further evidence that the mechanical integrity of the ILM may be lower in DD samples compared to non‐DD samples. The structural arrangement of collagen and elastin may be the key component responsible for the strength of cross‐bridges to limit AF deformation in the radial direction [18, 51, 52] and may also be the primary components associated with the four parameters related to the mechanical integrity of the ILM. Since no study has directly assessed the impact of changes in the composition of the ILM on the interlamellar properties, further studies are required for a better understanding of the role of specific macromolecules on the interlamellar properties.

Regional differences may also exist in the interlamellar properties of the AF. A previous study by Gregory et al. reported lower Peel Strength in the inner AF compared to the outer AF and trends for decreased Peel Stiffness of posterior AF compared to anterior AF [37]. Previous studies have reported similar trends comparing the differences between the inner AF and outer AF where the outer AF was reported to be stiffer [25]. Furthermore, higher amounts of cross‐bridges have been reported in the mid to outer AF compared to the inner AF in ovine discs [10]. However, the DD samples from the lateral regions did not meet the dimensional requirements required for peel tests as they were all 5 mm or under for the circumferential length. Furthermore, the DD samples were also mostly from the outer AF. Indirectly, these limitations allowed us to minimize the impact of regional variations on our results, allowing consistency in the interpretation of the differences between groups.

The present study had limitations that need to be acknowledged. First, as these studies were cross‐sectional, a cause‐and‐effect relationship between degeneration and mechanics could not be established. Samples were limited to DD individuals who underwent surgery, excluding cadaveric DD samples from nonsurgical cases. We also recognize that a longitudinal study with the current parameters to address this limitation is not feasible due to ethical restraints regarding human tissue collection. Second, heterogeneity in the degeneration of the AF tissue and the dimensions of the sample obtained from surgery could not be controlled, as most individuals were likely at different places on a disease progression trajectory. Consequently, our results from the outer anterior AF are not complete representations of the entire AF tissue mechanics and should not be extrapolated for the entire AF. Third, some samples were so severely degenerated (out of 65 possible samples, only 43 were tested, and 36 were included) that either they could not be mounted for testing or did not meet the minimum dimensional requirements. This led to individual selection bias, and the reported interlamellar properties from DD individuals may not reflect the range of interlamellar properties due to the exclusion of samples so severely degenerated as to not being suitable for mechanical assessment.

Future work will aim to increase the sample size to address any sex and lumbar level considerations to examine their effect on interlamellar properties. Furthermore, the cohort of diseased tissue will be expanded to include spine deformity individuals with degenerative scoliosis, degenerative spondylolisthesis, and isthmic spondylolisthesis to determine if different mechanisms may be involved in these different disease subsets. Weakening of the ILM could contribute to the decreased resistance to slippage between the lamellae and the development and progression of these deformities, and thus, the results would provide fuller insights into the contributions of the mechanical integrity of ILM in disease development and progression. Finally, future biochemical and immunolocalization studies will be integrated with the mechanical assessments to gain insights into potential cause‐and‐effect relationships.

## 5 Conclusions

The AF consists of concentric lamellae, and changes in the mechanical integrity of the ILM and cross‐bridges have been thought to play a role in the development of DD. The present study determined that all four interlamellar properties that were measured (Peel Stiffness, Peel Strength, Peel Toughness, and Standard Deviation of the Peel Stress in the Peel Region) were lower in surgical DD samples compared to the non‐DD samples. Overall, the present results indicate that the mechanical integrity of the ILM is compromised in fresh surgical DD samples compared to the corresponding tissues from non‐DD donors.

## Author Contributions

Study conceptualization and design: M.S.D., D.A.H., G.S., and N.A.D. Methodology: M.S.D., D.A.H., G.S., and N.A.D. Data collection and experiments: M.S.D. and M.A.S. Data analysis: M.S.D. Writing – original draft: M.S.D. Writing – review and editing: M.S.D., M.A.S., T.J.B., F.N., W.B.J., K.C.T., J.B., P.T.S., D.A.H., G.S., and N.A.D.

## Conflicts of Interest

The authors declare no conflicts of interest.

## Supporting information


**Table S1.** Basic demographic information of the Non‐DD and DD age‐matched individuals included in the study. Age, body mass index (BMI), and sex distribution are reported by the number of individuals, whereas the modified Pfirrmann Grade, radiographic degeneration grade, and disc level distribution are reported by the number of samples.
**Table S2.** Summary of the comparison of interlamellar peel properties between non‐DD and DD age‐matched individuals. Values are reported as mean ± standard deviation. *p ≤ 0.05 for statistical differences between non‐DD and DD age‐matched samples.
