# Supplementary material for: Human Disc Degeneration Is Accompanied by a Loss of Anterior Annulus Fibrosus Interlamellar Matrix Integrity as Assessed by Peel Tests
Source: JOR Spine. 2025 May 14;8(2):e70067. doi: 10.1002/jsp2.70067 (PMC12077525; doi:10.1002/jsp2.70067)
Supplement: Supplementary file 1 — Table S1. Basic demographic information of the Non‐DD and DD age‐matched individuals included in the study. Age, body mass index (BMI), and sex distribution are reported by the number of individuals, whereas the modified Pfirrmann Grade, radiographic degeneration grade, and disc level distribution are reported by the number of samples. Table S2. Summary of the comparison of interlamellar peel properties between non‐DD and DD age‐matched individuals. Values are reported as mean ± standard deviation. *p ≤ 0.05 for statistical differences between non‐DD and DD age‐matched samples. [file JSP2-8-e70067-s001.docx]

Differences in Mechanical Properties of the Annulus Fibrosus Interlamellar Matrix in Disc Degeneration

# Manmeet S. Dhiman^1^, Mohammed A. Salaam^1,2^, Taylor J. Bader^1,3^, Fred Nicholls^4^, W. Bradley Jacobs^4^, Kenneth C. Thomas^4^, Jacques Bouchard^4^, Paul T. Salo^1,4^, David A. Hart^1,4,5^, Ganesh Swamy^1,4^, Neil A. Duncan^1,6^

^1^McCaig Institute for Bone and Joint Health, University of Calgary, 3280 Hospital Dr NW, Calgary, Alberta T2N 4Z6, Canada

^2^Department of Biomedical Engineering, University of Calgary, 622 Collegiate Place NW, Calgary, Alberta T2N 4V8, Canada

^3^Department of Medical Sciences, University of Calgary, 3330 Hospital Dr NW, Calgary, Alberta T2N 4N1, Canada

^4^Department of Surgery, Cumming School of Medicine, University of Calgary, 1403 29 St NW,

Calgary, Alberta T2N 2T9, Canada

^5^Faculty of Kinesiology, University of Calgary, 376 Collegiate Blvd NW, Calgary, Alberta T2N 4V8, Canada

^6^Department of Civil Engineering, University of Calgary, 622 Collegiate Place NW, Calgary, Alberta T2N 4V8, Canada

*Supporting Information

Corresponding Author: Manmeet Dhiman, MSc (manmeet.dhiman@ucalgary.ca)

# **Supporting Information**

**Section S1 – Age-matched Analysis of the Data**

Table S1: Basic demographic information of the Non-DD and DD age-matched individuals included in the study. Age, body mass index (B.M.I.), and sex distribution are reported by the number of individuals whereas the modified Pfirrmann Grade, radiographic degeneration grade, and disc level distribution are reported by the number of samples.

|  | Non-DD | DD |
| --- | --- | --- |
| **Number of Individuals** | 7 | 7 |
| **Number of Samples** | 13 | 11 |
| **Age (years)** | 35 ± 9 | 37 ± 7 |
| **B.M.I.** | N/A | 27.7 ± 2.4 |
| **Modified Pfirrmann Grade** | N/A | 5.8 ± 1.2 |
| **Radiographic Degeneration Grade** | 0.25 ± 0.45 | N/A |
| **Sex (M/F)** | (5/2) | (5/2) |
| **Disc Level (L4-L5/L5-S1)** | (7/6) | (5/6) |

Table S2: Summary of the comparison of interlamellar peel properties between non-DD and DD age-matched individuals. Values are reported as mean ± standard deviation. * Indicates p ≤ 0.05 for statistical differences between non-DD and DD age-matched samples.

|  | **Non-DD** | **Age-matched DD** |
| --- | --- | --- |
| **Peel Stiffness (N/mm^2^)** | 0.27 ± 0.07 | 0.18 ± 0.05* |
| **Peel Strength (N/mm)** | 2.39 ± 0.93 | 1.37 ± 0.49* |
| **Peel Toughness (J/m)** | 34.33 ± 15.05 | 16.83 ± 8.70* |
| **Standard Deviation of the Peel Region (N/mm)** | 0.076 ± 0.045 | 0.044 ± 0.028* |
